# Analyzing Moment Arm Profiles in a Full-Muscle Rat Hindlimb Model†

**DOI:** 10.3390/biomimetics4010010

**Published:** 2019-01-25

**Authors:** Fletcher Young, Christian Rode, Alex Hunt, Roger Quinn

**Affiliations:** 1 Department of Mechanical and Aerospace Engineering, Case Western Reserve University, Cleveland, OH 44106-7222, USA; rdq@case.edu; 2 Department of Motion Science, Friedrich-Schiller University Jena, 07737 Jena, Germany; christian.rode@uni-jena.de; 3 Department of Mechanical and Materials Engineering, Portland State University, Portland, OR 97207, USA; ajh26@pdx.edu

**Keywords:** biomechanics, moment arm, biarticular muscles

## Abstract

Understanding the kinematics of a hindlimb model is a fundamental aspect of modeling coordinated locomotion. This work describes the development process of a rat hindlimb model that contains a complete muscular system and incorporates physiological walking data to examine realistic muscle movements during a step cycle. Moment arm profiles for selected muscles are analyzed and presented as the first steps to calculating torque generation at hindlimb joints. A technique for calculating muscle moment arms from muscle attachment points in a three-dimensional (3D) space has been established. This model accounts for the configuration of adjacent joints, a critical aspect of biarticular moment arm analysis that must be considered when calculating joint torque. Moment arm profiles from isolated muscle motions are compared to two existing models. The dependence of biarticular muscle’s moment arms on the configuration of the adjacent joint is a critical aspect of moment arm analysis that must be considered when calculating joint torque. The variability in moment arm profiles suggests changes in muscle function during a step.

## 1. Introduction

In recent years, models of multimuscled systems have sought to replicate both the kinematics and control regimes of living organisms. These models are now being used to explore the challenges of coordinating many actuators to move a relatively small number of joints. Understanding how muscled organisms rapidly coordinate overactuated systems to accomplish tasks could suggest new design frameworks for the robotics community. The agility and elegance of even the simplest muscled organism is an enviable benchmark for robotics, which struggle to emulate the power and adaptability of animals. Modeling the kinematics of a multimuscle system and its associated neurological control signals is a complex task that requires an understanding of both engineering and biological principles.

Modeling muscle coordination could provide insight into the impact and evolutionary incentive for utilizing overactuated systems. The overabundance of muscles with respect to the number of degrees of freedom has been a focus of research for many years. Muscle redundancy has long been characterized as a defense mechanism to preserve limb actuation in the case of individual muscle failure. Recently, this assumption has pivoted from less of a physiological imperative to more of a task-specific evolutionary development [1,2]. Muscle redundancy may be more of an evolutionary adaptation for carrying out a wide range of activities, as well as a safeguard against injury. 

This idea is in line with studies that characterize muscles based on task-driven physiological parameters called muscle synergies [3,4,5]. These synergies have been shown to reduce the computational burden on the nervous system by organizing muscle activity into functional groups. Rather than the nervous system coordinating the activity of each muscle individually, higher-level processing units may direct muscle groups to carry out abstract limb motion to accomplish a goal. Further research is needed to better understand the kinematic and neurological implications of multimuscle coordination.

Previous work by Hunt et al. [6] has produced a three-dimensional (3D) model of a rat skeleton with muscle-driven locomotion controlled by modeled motorneuron signals. This model is implemented in AnimatLab [7], software that simulates both a 3D physics environment and a neural design environment. AnimatLab allows users to simultaneously monitor the biomechanical properties of simulated animals while also providing insight into the neurophysiological connections associated with locomotion.

Hunt’s model abstracts rat walking through the contraction of monoarticular muscles which flex and extend the hindlimb joints. The model demonstrates the capability of “deconstructing” an organism’s joint kinematics into modeled motorneuron signals, which can then act as the drivers for artificial locomotion. Here, we expand upon the model by incorporating a complete muscular system, including biarticular muscles that span two joints.

Accounting for biarticular muscles is mandatory for understanding physiological locomotion in rodents. For example, in the rat, the average cross-sectional area of biarticular muscles is about 33% larger than for monoarticular muscles [8]. Human leg models have also shown that biarticular muscles play a critical role in the generation of forces that monoarticular muscles lack [9,10,11]. Therefore, including these muscles, their forces, and their effect on joint torques is critical to understanding nervous system control of locomotion.

Joint torque calculations depend both on accurate muscle force modeling as well as robust moment arm profiles. Calculating muscle moment arms is a complex task, as muscle paths are seldom unidirectional and different analytical methods can yield varied results [12,13,14,15]. Moment arm calculations have been shown to affect error in a force prediction model [16]. A technique rooted in biomechanical fundamentals is necessary to understand the varied nature of moment arm profiles during walking.

Muscle moment arm profiles in static hindlimb rat models [17] and in the mouse hindlimb [18] have been developed. In these works, moment arms are calculated by fixing all joints except one, and moving that joint through its range of motion. In combination with joint angle data from a step, these data can be used to analyze moment arms of monoarticular muscles in locomotion. However, to consider the joints in isolation is to ignore the dependency of the function of biarticular muscles on the coordinated motion of adjacent joints. The moment arms and resultant torque of biarticular muscles of one joint depend on the motion of the other joint. Moreover, biarticular muscle length changes and, thus, their dynamics also depend on the coordinated motion of adjacent joints [19]. This methodology is therefore not sufficient for analyzing the complete physiological locomotion.

This work describes a methodology rooted in fundamental principles to generate moment arm profiles from muscle attachment points for use in analyzing physiological locomotion. Specifically, this method is well suited for analyzing moment arms of biarticular muscles, muscles which wrap around joints, and muscles that contain multiple via points. In contrast to the single-joint analysis of the comparative models, the work includes the effects of simultaneous multijoint motion during a normalized step cycle, producing the physiologically relevant muscle moment arms as the hindlimb moves through stepping. This method is validated against two existing hindlimb models by comparing the moment arm profiles of single-joint motion. Finally, 3D moment arm profiles for multijoint motion are developed and examined.

## 2. Materials and Methods

### 2.1. Hindlimb Bone Segments

**Figure 1:**
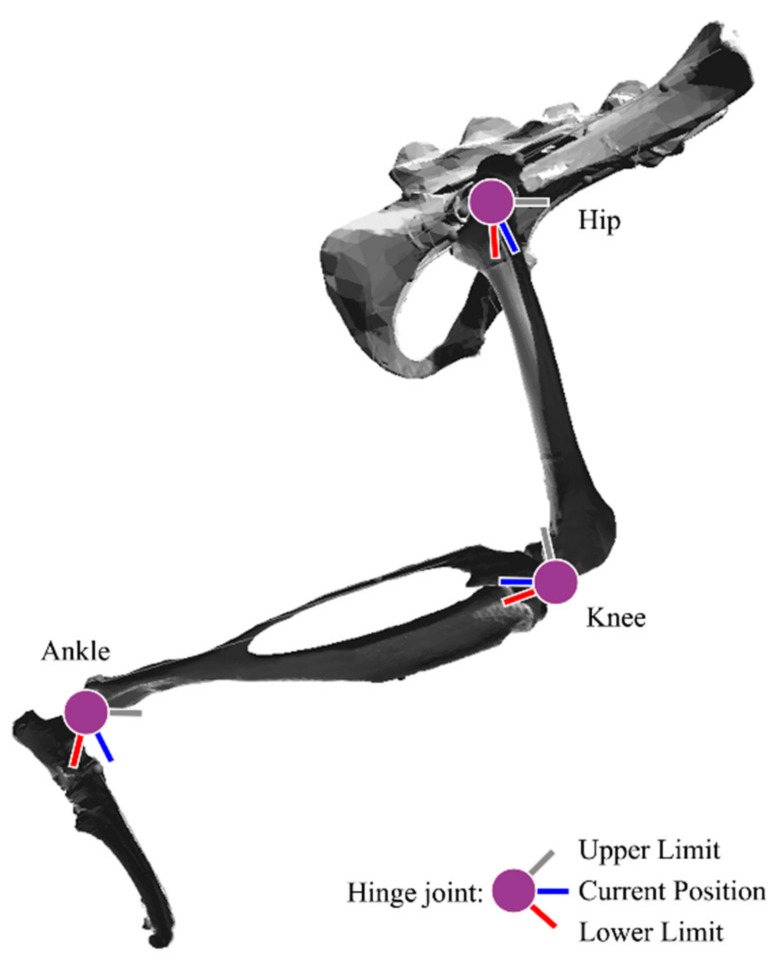
Hindlimb bones segments from *Rattus norvegicus*. Hinge joints are represented at the hip, knee, and ankle. The absolute joint angle limits are represented by the red and gray bars, while the blue bars represent the current orientation of the distal body.

Four hindlimb segments had previously been scanned from the bones of a brown rat, *Rattus norvegicus* [6]. The pelvis, femur, tibia, and foot of the hindlimb were articulated using hinge joints, as shown in Figure 1. Hinge joints allow the connected segments to move through a range of flexion and extension, allowing for the application of sagittal plane motion from X-ray video analysis of parasagittal plane locomotion. For this analysis, only sagittal plane motion was considered, resulting in three degrees of freedom. 

Joint centers were placed using similar methods to both Johnson et al. [17] and Charles et al. [18]. The hip joint was placed such that the femoral head rested within the acetabulum. The knee joint was placed between the condyles of the femur such that the tibia and femur do not collide within the joint’s range of motion. The ankle joint was placed between the tibial malleoli, proximal to the calcaneus. 

Joint angle limits were determined from the work of Fischer et al. [20]. Centers of rotation are stationary relative to the reference frame of the distal body and, therefore, do not undergo relative translation during walking. As motion is considered only in the sagittal plane, all joint axes remain parallel during motion. 

### 2.2. Anatomically Derived Muscle Paths

The addition of muscles to the model was guided, in part, by 3D data gathered from anatomical dissections [17] as well as from Greene’s primer on rat anatomy [21]. The *xyz* muscle attachment coordinates from Johnson et al. [17] served as a baseline approximation for the attachment area. Due to the differences in scanned bone sizes and the misalignment of bone-centric coordinate systems, utilizing the *xyz* data directly was not sufficient for generating a physiologically plausible model.

AnimatLab does not restrict muscle pass-through on bone structures, allowing muscles to pass completely through bone during the walking cycle. Moreover, AnimatLab does not have built-in capabilities for muscle wrapping. To make a physiologically relevant model, the muscles were guided from origin to insertion using via points along paths that avoid bone pass-through according to the descriptions and figures from Greene. Special care was taken to guide muscles around joints and insert them onto structures that were physiologically similar to their real-life counterparts. No effort was made to avoid muscle pass-through.

**Figure 2:**
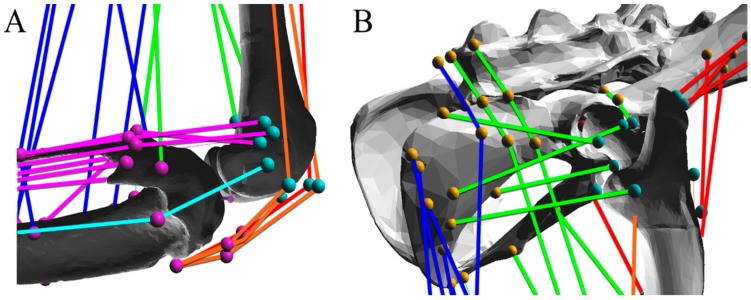
Muscle paths were determined based on anatomical descriptions and diagrams from Greene [21]. Enlargements of (**A**) the knee and (**B**) hip detail the complex interconnection of muscle attachment points. Attachment points are stationary within local bone coordinate systems. Attachment points are carefully placed such that there is no bone pass-through during the physiological representative walking cycle.

**Figure 3:**
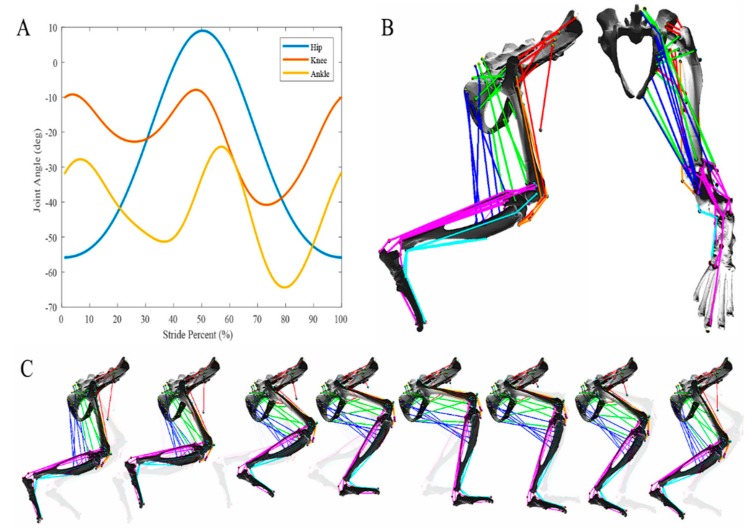
A physiologically relevant walking cycle has been implemented in the AnimatLab simulation. (**A**) Joint angle profiles were developed from walking data [20]. Joint angles are relative to the zero-angle convention established in the AnimatLab simulation, based on the geometric configuration of the joints. (**B**) Complete musculature of the hindlimb from the sagittal and rear coronal view. Coloration added only for visual contrast. (**C**) Complete walking cycle demonstrating the musculature relative to the bone structures during walking. Semitransparent leg configurations included representing the previous leg configuration.

The colored muscle paths in Figure 2 and Figure 3 represent muscle lines of action. Attachment points are shown as single points representing the centralized attachment area for muscles. Coloring is included to aid in model design, and muscles are sorted based on their general muscle activity, although this colorization has no impact on muscle parameters or moment arm profiles.

### 2.3. A Physiologically Representative Walking Profile

X-ray video analysis of a walking rat was used to generate sagittal plane motion of the hip, knee, and ankle for a normalized stride period during walking on a flat plane as reported elsewhere [20,22,23]. Average joint motion profiles from X-ray data were decomposed into sum-of-sines equations in MATLAB (MATLAB 2017b, The MathWorks, Inc., Natick, MA, USA), then implemented in AnimatLab. In AnimatLab, a joint angle motor induces the motion according to the acquired equation. Application of joint motion directly into the 3D environment allows for muscle motion analysis during multijoint stepping motions, aiding in the analysis of muscle movement with respect to hindlimb segments.

### 2.4. Calculating Muscle Moment Arms

**Figure 4:**
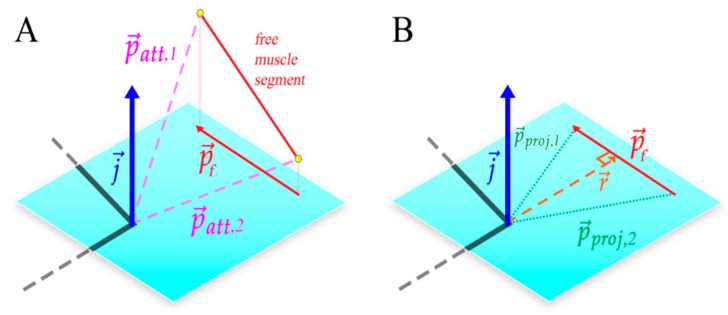
Calculating the muscle moment arm, *r.* (**A**) The plane of interest and its coordinate system is defined by the joint center and the joint axis representing flexion/extension (blue). Joint axes are defined using the same convention as Johnson et al. [17] and Charles et al. [18]. Orthogonal joint axes represent abduction/adduction, and inversion/eversion. (**B**) The free muscle segment that connects the adjacent bone segments (monoarticular muscles) or to the bone segment after the next (biarticular muscles) is projected onto the plane of interest. This projected free segment is called ${\vec{p}}_{f}$. The muscle moment arm *r*, is calculated from the joint axis $\vec{j}$ and ${\vec{p}}_{f}$.

A muscle moment arm describes the distance of the muscle line of action from a joint axis, as shown in Figure 4. This distance is critical to analyzing the muscle’s ability to generate torque about the joint axis. For example, the biceps femoris posterior can generate a relatively large amount of torque about the knee with little force because of its large moment arm.

In AnimatLab, muscle attachment points remain stationary relative to the bones they are attached to. For this reason, moment arms for multisegment muscles (muscles that wrap around structures and have more than two attachment points) are solely dependent on the free muscle segment spanning the joint(s) that undergoes a length change over joint motion. Moment arm measurements are taken at discrete times during motion.

To calculate the moment arm of a muscle segment, the muscle attachment points of the free muscle segment ${\vec{p}}_{att,i}$ are projected onto the plane of interest defined by the joint axis $\vec{j}$ such that

(1) $${\vec{p}}_{proj,i}={\vec{p}}_{att,i}-\frac{{\vec{p}}_{att,i}\cdot \vec{j}}{|\vec{j}{|}^{2}}\vec{j}.$$

Muscle segments are represented by the subtraction of projection attachment positions of the free segment, creating the projected muscle segment vector ${\vec{p}}_{f}$:

(2) $${\vec{p}}_{f}={\vec{p}}_{proj,i+1}-{\vec{p}}_{proj,i}.$$

The projected muscle segment is then crossed with the joint axis to determine the moment arm’s direction. The sign of the dot product between a projected muscle attachment path ${\vec{p}}_{proj,i}$ and the moment arm determines whether the muscle is inducing positive or negative joint motion. The final moment arm length is represented as the scalar value r:

(3) $$r={\vec{p}}_{proj,1}\cdot \frac{{\vec{p}}_{f}\times \vec{j}}{\left||{\vec{p}}_{f}\times \vec{j}|\right|}.$$

### 2.5. Sensitivity Analysis

To determine the impact of that muscle attachment geometry has on the moment arm profiles, a sensitivity analysis was performed. For the isolated joint motion simulation, each free muscle segment attachment point was moved by 1 mm independently, and the moment arm profile was calculated. The moment arm profiles for the four attachment movements are examined.

For the biceps femoris anterior, the proximal attachment was moved cranially and caudally along the body of the ischium, and the distal attachment was moved proximally and distally along the lateral condyle of the tibia. The proximal attachment of the pectineus was moved cranially/caudally along the acetabulum, and the distal attachment was moved proximally/distally along the linea aspera of the femur. The proximal attachment of the semimembranosus was moved dorsally/ventrally along the body of the ischium, and the distal attachment was moved proximally/distally along the dorsomedial ridge of the tibia.

The proximal attachment of the vastus intermedius was moved proximally/distally along the line of the femur and the distal attachment was moved proximally/distally across the surface of the tibial tuberosity. The proximal attachment of the medial gastrocnemius was moved proximally/distally along the tibial line of action and the distal attachment was moved dorsally/ventrally along the posterior calcaneus of the foot. The proximal attachment of the tibialis anterior was moved proximally/distally along the extensor surface of the tibia and the distal attachment was moved proximally/distally along the dorsiflexor surface of the foot. 

## 3. Results

**Figure 5:**
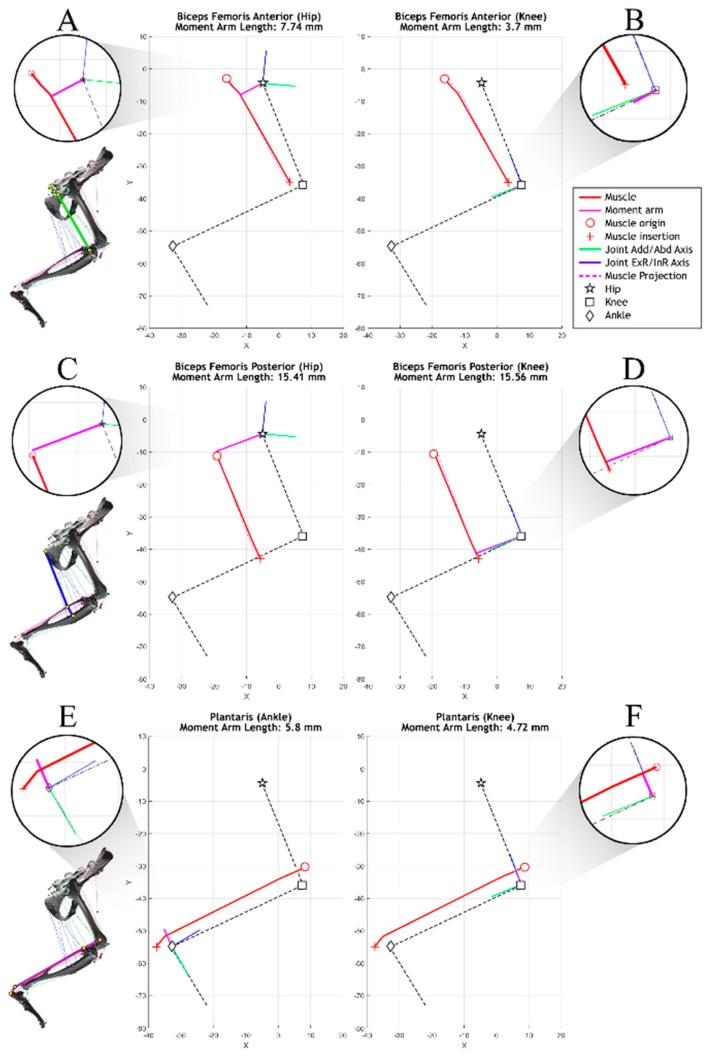
Simulating moment arms for three biarticular muscles including (**A**,**B**) the biceps femoris anterior, (**C**,**D**) biceps femoris posterior, and (**E**,**F**) plantaris. Moment arms (purple) are calculated about each of the spanned joints. Muscle paths are highlighted on the 3D figures on the left with flyouts showing detail about the joint space. Scalar moment arm lengths are derived from these simulations in the sagittal plane. External/internal rotational axes (ExR/InR), represented in blue, and adduction/abduction axes (Add/Abd), represented in green, are shown but not presently analyzed.

The moment arms of three biarticular muscles are shown in Figure 5, demonstrating the impact that joint selection and leg configuration has on moment length calculations. As part of model validation, single-joint articulation was compared to moment arm data from Johnson et al. [17] and Charles et al. [18]. These works focus on moment arm generation in static models of the mouse and rat hindlimb, respectively. Models in these studies were moved through a much larger range of motion than that typically seen in rat walking and the data has, therefore, been truncated to match the physiological joint ranges studied in this model. The associated models do not include multijoint motion, an important characteristic for determining the muscle moment arms of biarticular muscles.

**Figure 6:**
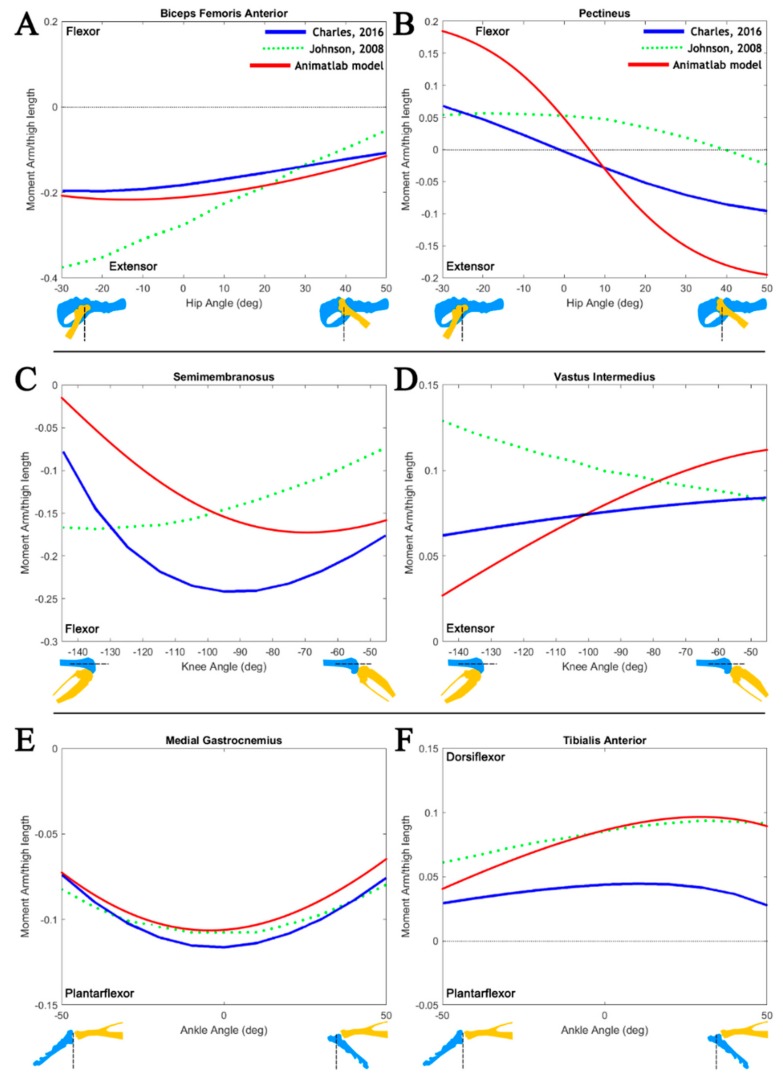
Comparison of hindlimb moment arms calculated using the AnimatLab model to data from Johnson et al. [17] and Charles et al. [18] (data adopted from [18], published under the Creative Commons Attribution (CC BY) license [24]). Moment arm profiles were developed for muscles under single-joint motion of (**A**,**B**) the hip, (**C**,**D**) knee, and (**E**,**F**) ankle. Joint angles follow the zero-angle convention established in the existing models. Joint configurations (yellow/blue) are provided for reference with the zero-angle noted. *y*-Axes are moment arm lengths normalized to the model’s femur length.

Figure 6 shows the relative moment arm sizes for the range specified, along with the joint of interest and the range of motion. For proper size comparison, the moment arms have been scaled to the femur lengths of the respective animals (mouse = 16.25 mm [18], rat = 35.00 mm [17], AnimatLab model = 35.75 mm). Joints that are not in motion during moment arm analysis are held at zero-angle as specified in [17] and [18]. Noteworthy, these moment arm profiles lack the influence of biarticularity on the selected muscles.

**Figure 7:**
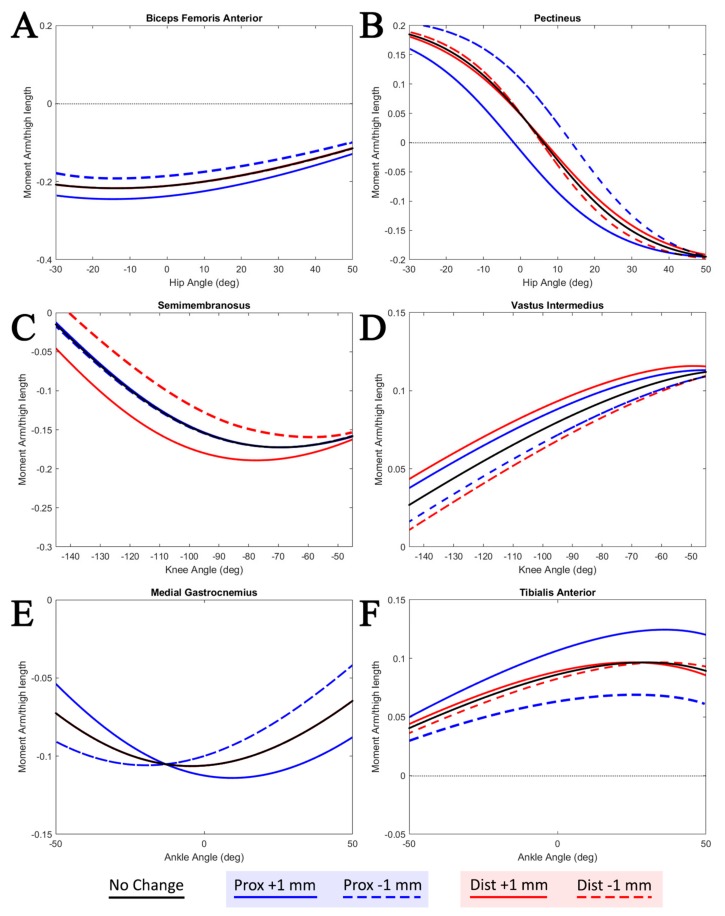
Sensitivity analysis of the moment arm profiles for muscles about (**A,B**) the hip, (**C,D**) knee, and (**E,F**) ankle. Proximal (prox) and distal (dist) attachment points for the free muscle segment are individually moved ±1 mm, and the moment arm profile is calculated. Moment arm profiles are recorded under the same isolated joint motion presented in Figure 6. *y*-Axes are moment arm lengths normalized to the model’s femur length.

Figure 7 shows the sensitivity analysis of the moment arm profiles as the free muscle segment attachment points are moved. In general, the moment arm profile is most sensitive to the movement of the attachment point closest to the joint.

**Figure 8:**
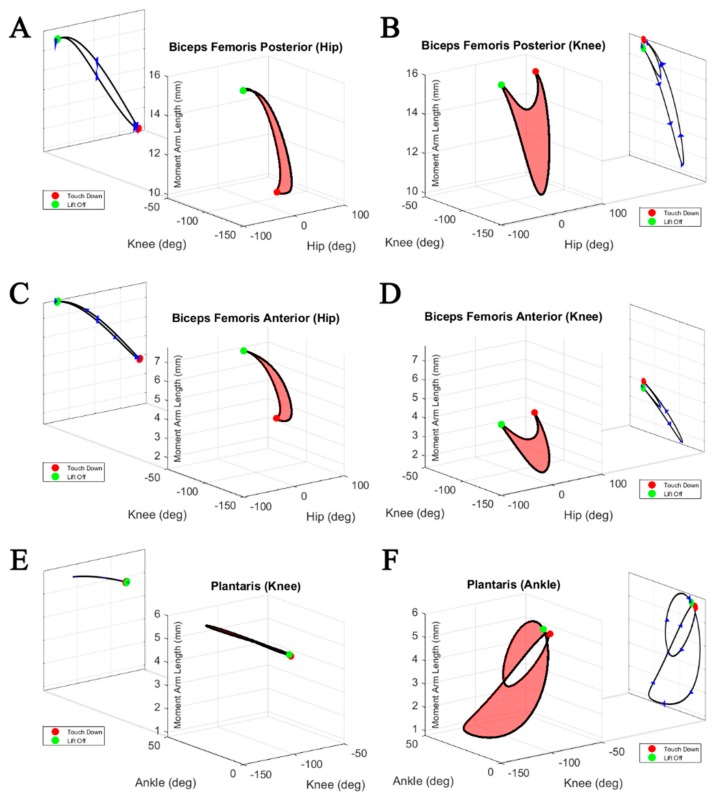
Moment arm profiles of (**A,B**) the biceps femoris posterior, (**C,D**) biceps femoris anterior, and (**E,F**) plantaris as calculated about their spanned joints over a physiological walking profile. Muscle moment arm length travels along the outside of the surface over the step cycle. Projections of the profile are shown to the side, representing the profile shape for a single joint motion. Arrows indicate the development of the moment arm over the step cycle, and colored dots represent touch down (red) and lift off (green). Joint angles follow the zero-angle conventions established by Johnson et al. [17] and Charles et al. [18].

Figure 8 shows the cyclical moment arm profiles of biarticular muscles under the action of a physiological stepping motion. The red surfaces represent the range of moment arm lengths reachable within the bounds of physiological walking. These moment arm profiles demonstrate the extreme variability of muscle moment arms for different joint angles. Using the moment arm profile as a simple lookup table for finding the semitendinosus accessory moment arm about the knee at −30 degrees, for example, could yield two different moment arm lengths that differ by about 35%.

Not only are moment arm profiles of individual muscles unique among muscles of similar action, but moment arm profiles are different about the same rotational axes of different joints. In Figure 8, the three biarticular muscle moment arm profiles are shown with respect to their two joints of impact. When analyzing the moment arm about the knee, the biceps femoris anterior generates moment arms within the range of 1.5–4.0 mm. By contrast, about the hip the same muscle generates moment arms within the range of 3.7–7.7 mm. The range of moment arms of the plantaris about the ankle varies from 6 to 1 mm, while it remains almost constant at about 6 mm at the knee.

**Figure 9:**
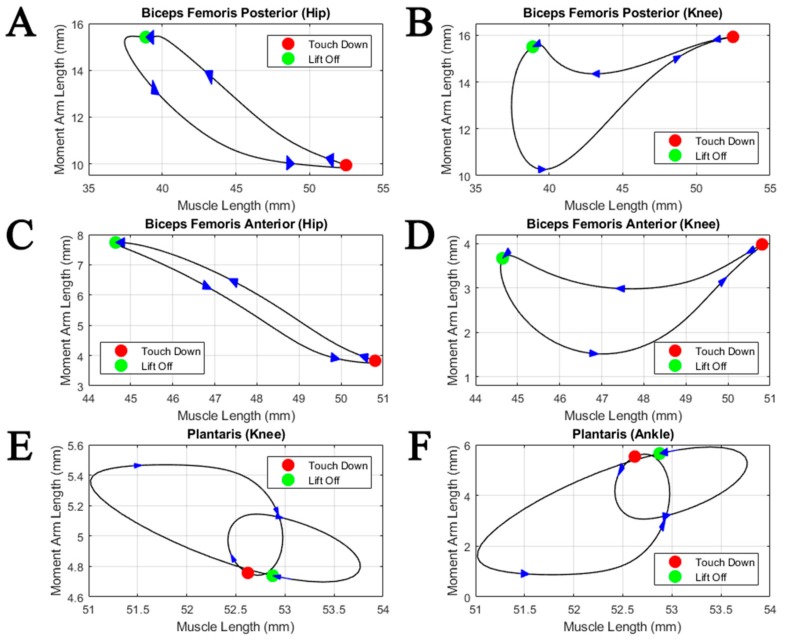
Biarticular moment arm profiles with respect to muscle length during simultaneous multijoint motion under a physiological walking pattern. Two moment arm profiles are provided for (**A,B**) the biceps femoris posterior, (**C,D**) biceps femoris anterior, and (**E,F**) plantaris, representing analysis about either spanned joint. Arrows indicate increasing step cycle direction (from stance to swing) with equal time spacing, and colored dots represent touch down (red) and lift off (green).

Normalized biarticular moment arm profiles are plotted against their normalized muscle lengths in Figure 9. Muscle length coupled with the moment arm length can be used to infer torque directions during the step cycle. During the stance phase, the hip to knee moment arm ratios change from 0.6 to 1 for the biceps femoris posterior, compared to a change from 1 to 2 for the biceps femoris anterior. Interestingly, at the same time, the moment arms at the knee remain rather constant for all shown muscles. Both biceps femoris posterior and anterior show pronounced shortening during the stance phase, while the length of the plantaris remains constant.

## 4. Discussion

This work describes the development process for a model of the rat hindlimb with a complete set of muscles. It expands upon previous models that have linked the kinematics of the hindlimb to the nervous system with the intent of understanding the interactivity of the nervous system and the musculature. An expanded muscle model provides the fundamental framework for future intermuscle coordination for limb motion. In addition to the physical makeup of the leg, a physiological walking process has been adapted from existing work in order to analyze the moment arms of each muscle.

The sensitivity analysis results shown in Figure 7 demonstrate the impact that muscle attachments can have on moment arm calculations. Trends in the muscle attachment motions provide insight into the possible differences between the associated models. Moving the proximal attachment point of the pectineus caudally along the pelvis shifts the functional transition angle (angle at which the muscle changes from a flexor to extensor) more negative, similar to the transition angle demonstrated by Charles et al. [18]. Moving the distal attachment of the semimembranosus distally along the tibia has a similar effect.

While the attachment point motion is capable of describing some differences in the model, there are overall trends which are different. As the knee extends, the AnimatLab moment arm profile increases. A similar but less dramatic trend is seen in the model by Charles et al. [18], but the trend is reversed in the model by Johnson et al. [17]. This could be the result of the distal attachment point of the vastus intermedius moved further along the length of the femur or the inclusion of the distal attachment point in the tibial reference frame.

Simplifying muscle geometry to a single path is an effective method for calculating moment arm lengths, but abstracts away from the complex muscular environment of the hindlimb. Broad muscles with long insertion lines (e.g., the biceps femoris muscle) are involved in a complex array of joint motion, making it difficult to identify a single moment arm length to represent the entire muscle. For this reason, larger muscles have been separated into multiple lines of actions. This method is not a perfect representation of a muscle’s torque-generating capabilities, but is an adequate method for exploring the impact of neural-controlled muscular force generation in a simulated modeled environment. In future, the simulation could be expanded by including more lines of action or generalizing the muscle insertions in such a way as to accurately simulate the impact that broad muscles have on torque generation.

The location of the joint center has a large impact on the calculation of a muscle moment arms since their length is defined relative to the position of the joint. Hinge joints in the rat are the result of the articulation of nonspherical bone surfaces which cause the joint center to migrate about articular surfaces during motion. As such, muscle moment arm lengths can change during locomotion by the motion of the joint center alone. The manual placement of joint centers applies an inherent simplification into the model which abstracts away the complicated nature of joint motion. This simplification is the result of software limitations wherein the joint center maintains a constant position relative to the articulating surfaces. In future, a more detailed version would incorporate a moving joint center to capture its impact on 3D muscle moment arm profiles.

Isolated joint motion, while limited in scope, can provide some insight into muscle function. Figure 6 compares isolated joint muscle moment arms against two existing hindlimb models. This information can be used as a litmus test to infer which (if any) limb motion (flexion/extension, abduction/adduction, etc.) the muscle is most likely to impact. For example, the isolated joint moment arm profile for the pectineus shows that the muscle switches from a hip extensor to a hip flexor as the joint extends. This transition in muscle function is unique among monoarticular muscles in the model.

Most monoarticular muscles have a single primary action while walking. The tibialis anterior, a muscle that runs along the length of the tibia and inserts into the foot, acts solely as an ankle dorsiflexor. Since muscles are only capable of generating contractile tension along their lines of action, the muscle moment arm is the definitive factor in the directionality of torque applied to a joint. Modeling torque generation accurately in a hindlimb model is a fundamental step toward generating the proper control signals for coordinated, muscle-driven locomotion.

By contrast, most biarticular muscles serve multiple roles. Rats seldom move a single joint in their hindlimb while walking, which makes it necessary to consider biarticular moment arm length changes induced by both spanned joints. Figure 8 shows 3D surfaces representing viable moment arm lengths as the spanned joint angles change during physiological walking. For most monoarticular muscles, like the tensor fascia latae and tibialis posterior, the moment arm profile is almost completely coupled to the joint of interest. Conversely, muscles like the biceps femoris posterior and the plantaris are dramatically affected by both joints that they span. To determine the correct moment arm length, it is necessary to know the configuration of both associated joints.

The variability in the moment arm and length change profiles (Figure 8 and Figure 9) indicates different, and maybe changing, functions of the muscles during the step. Previous studies of biarticular muscle function often relied on the simplifying assumption of constant muscle moment arms [25,26]. The extremely varied moment arm profile of the plantaris about the ankle (Figure 8) shows that, while always acting as an extensor, ankle extension may rely on the assistance of other muscle contractions during times when the plantaris’ moment arm is short. Considering their pronounced shortening throughout the stance phase, the biceps femoris posterior and anterior could be used as motors, while the rather constant length of the plantaris suggests an energy transferring function (“ligamentous action” [27]) resulting in synchronized joint movements. These distinctions could provide insight into how the nervous system prioritizes motorneuron activation when inducing motion.

## 5. Conclusions

We developed a method that uses vector analysis to calculate moment arms during physiological walking. Moment arms for a set of muscles were compared to that in the literature, demonstrating comparable moment arm ranges (Figure 6) to existing hindlimb muscle models. In addition to single-joint model validation, this work demonstrates the importance of multijoint moment arm analysis. Moment arm profiles which capture the effects of simultaneous multijoint action demonstrate the complex roles that muscles can assume while walking. Future analysis will expand upon our planar model by incorporating additional degrees of freedom at the hip and ankle.

Joint torque calculations coupled with moment arm profiles can be used to determine muscle forces through a distribution scheme. Once this distribution scheme is developed, activation curves for individual muscles can be developed, replicating the torque data observed from walking animals. Understanding how the kinematics of the system manifest in the model is essential to developing a complete neuromechanical model of rat locomotion that utilizes physiologically relevant data to understand neural control and redundant muscle coordination.
